# Rapeseed-based diet modulates the imputed functions of gut microbiome in growing-finishing pigs

**DOI:** 10.1038/s41598-020-66364-4

**Published:** 2020-06-10

**Authors:** Özgün Candan Onarman Umu, Liv Torunn Mydland, Margareth Øverland, Charles McLean Press, Henning Sørum

**Affiliations:** 1 Department of Paraclinical Sciences, Faculty of Veterinary Medicine, Norwegian University of Life Sciences, P.O. Box 369, N-0102 Oslo, Norway; 2 Department of Animal and Aquacultural Sciences, Faculty of Biosciences, Norwegian University of Life Sciences, P.O. Box 5003, N-1432 Ås, Norway; 3 Department of Preclinical Sciences and Pathology, Faculty of Veterinary Medicine, Norwegian University of Life Sciences, P.O. Box 369, N-0102 Oslo, Norway

**Keywords:** Bacteria, Microbial communities

## Abstract

Rapeseed meal is a sustainable feed ingredient that can be used as an alternative to imported soybean meal in European pig production. The gut microbiota plays an important role on pig physiology and health but the impact on microbiota of using rapeseed in diets is still not well known. In this study, 84 purebred Norwegian Landrace pigs with average initial weight of 25 kg were divided into two groups and fed for approximately three months with either a control diet containing soybean meal (CON) or a high-fiber experimental diet where 20% rapeseed meal (RSF) was included as an alternative to soybean meal in CON. The composition and function of microbiome in gut digesta samples were analyzed by performing 16S *rRNA* gene sequencing and culturing of bacteria. The microbiota diversity and composition were similar between the dietary treatments; however, relative abundance of a variety of bacterial groups and imputed functions of microbiome in the ileum and large intestine were altered when the pigs were fed with a rapeseed-based diet. It was notable that the immune-inducing bacterial group *Mucispirillum* and anti-inflammatory stimulating bacteria *Lachnospira* were more abundant in the ileum and large intestine of the RSF group, respectively. Moreover, there was a higher abundance of major amino acid fermenters and amylolytic bacteria in the CON group and a high abundance of putative short chain fatty acid producers in RSF group. In comparison with the CON group, the gut microbiome of RSF group possessed an enhanced potential for carbohydrate and energy metabolism and a reduced potential for bacterial pathogenicity-related pathways.

## Introduction

Rapeseed is a locally produced and sustainable feed ingredient that has the potential to be an alternative protein source to imported soybean meal in diets used in European pig production industry^1^. Rapeseed meal contains anti-nutrient components such as glucosinolates, sinapine, tannins, and erucic acid and higher amount of dietary fiber compared with soybean meal that is conventionally used in pig diets. Thus use of rapeseed in pig diets may lead to a reduction in nutrient utilization, feed intake and growth rate^2,3^. On the other hand, dietary fibers act as a substrate for gut microbiota in the host and may affect the host favorably, contributing to maintenance of gut health and enhancement of general well-being^4–6^.

Growth performance related parameters have mostly been considered the major parameters in animal production, in terms of evaluation of alternative feed ingredients. However, the increasing demand for improvement of animal welfare and environmental issues entails having a globally responsible and sustainable system with a focus on animal health. In this respect, a better understanding is needed of the response of gut microbiota to alternative feed components to evaluate the impact of feed components on the host physiology and health.

A limited number of studies has been conducted to investigate the effect of rapeseed meal on gut microbiota populations compared with soybean-based diet, while, to our knowledge, no study has reported the functional response of microbiota to rapeseed as a feed ingredient. In one study, higher *Lactobacillus* spp. in caecum and lower *Clostridium perfringens* in the mid-colon were found based on culturing when soybean meal was replaced by rapeseed meal in the diet of growing-finishing pigs^7^. Moreover, based on 16S *rRNA* gene sequencing and culturing methods, we have previously found no change on diversity or composition of gut microbiota in weaner pigs when soybean meal was replaced with rapeseed meal and hulls in the diet of pigs^8^. On the other hand, some opportunistic pathogenic groups, including *C. perfringens*, were less abundant, while colon health- and immune system-associated bacterial populations, such as *Dialister*, *Lachnospira*, *Coprococcus*, *Shuttleworthia*, unclassified Erysipelotrichaceae and unclassified Clostridiales, unclassified Coriobacteriaceae, were more abundant in RSF-fed weaner pigs^8^. These findings suggested that the high-fiber rapeseed-based diet modulated the gut microbiota in a beneficial way in weaner pigs^8^. In this study, as a follow-up, our objective was to assess the effects of RSF on the gut microbiome composition of growing-finishing pigs by performing both 16S *rRNA* gene sequencing and culturing of specific bacterial groups. We also predicted the imputed functions of microbiome to examine how the gut microbiome function of growing-finishing pigs respond to RSF compared with CON.

## Materials and Methods

### Animals, feeding and experimental design

This study is a part of an extensive experiment on growing-finishing pigs, where the fiber-rich diet effect on function of different organs, intestinal mucosa, epithelial barrier, gut microbiota and immune homeostasis was investigated *in vivo* after a three-month experimental feeding period. In this study, total of 84 purebred Norwegian Landrace pigs (average initial body weight 25 ± 2 kg) from 16 litters were divided into two dietary treatment groups (n = 42 per group) by initial weight with an equal number of males and females. The experiment was conducted as a randomized complete block design. The pigs were blocked by litter and sex and distributed into fourteen pens with six pigs in each pen. The straw bedding in pens was omitted due to the risk of affecting the nutrient digestibility and gut microbiota of pigs, thus instead all pens were equipped with rubber mats and activity enrichment tools. The experiment was conducted at the Center for Animal Research, Norwegian University of Life Sciences, Aas, Norway. The experimental protocol for the study was approved by the Norwegian Food Safety Authority (ID: 8217) such that the experiment was performed in accordance with relevant guidelines and regulations.

The diets used in the experiment were formulated to meet the energy and nutrient requirements for growing-finishing Landrace pigs. The soybean-based control diet (CON) formulation was a typical commercial-like Norwegian diet for growing-finishing pigs. The experimental diet RSF contained 20% rapeseed meal, replacing all soybean meal and a small proportion of the barley and wheat in CON (Table 1). The diets were designed to be isoenergetic and isonitrogenous and to contain equal levels of standardized ileal digestible (SID) amino acids. Crystalline amino acids were used to adjust the SID lysine on net energy basis. Diets were formulated to meet or exceed the requirements for indispensable amino acids and all other nutrients. A cumulative feed sample from each dietary treatment was subjected to chemical analysis^2^. Animals were individually fed twice a day according to a semi-*ad libitum* feeding regime. The amount of feed to be given to the animals was adjusted on a weekly basis according to individual appetite. Individual feed intake was recorded, and feed leftovers were noted. Animals had free access to clean drinking water in pens. More details on the animals, feeding and the experimental design are described by Skugor *et al*.^2^.

**Table 1:** Dietary composition of the experimental diets.

Ingredients (g kg^−1^)	Diet	
	CON	RSF
Barley	380.2	340.4
Wheat	240.0	233.4
Oats	140.0	140.0
Soybean meal (45% CP)	150.0	0.0
Rapeseed meal	0.0	200.0
Rendered fat (tallow)	50.4	50.0
Supplements^a^	39.4	36.2

^a^Minerals, amino acids, vitamins, and yttrium oxide (more details in Skugor *et al*. 2019).

Each pig was fed 2–2.5 hours before slaughter. Pigs were fixed and slaughtered using head-to-heart electrical immobilization, followed by exsanguination at a mobile slaughterhouse (Mobilslakt AS, Norway). The intestinal contents from the ileum, caecum and colon were collected. Samples were put into tubes containing saline solution for culturing and/or were snap-frozen and kept at −80 °C until processing for the microbiota analysis. The ileum and colon samples from all 84 animals were used for culturing, while the ileum, caecum and colon samples from 48 of 84 animals (n = 24 per treatment group) were processed for 16S *rRNA* gene sequencing. The 48 pigs for sequencing were selected based on the feed conversion ratio (FCR) including 12 pigs with FCR higher than the average and 12 with FCR lower than the average for each dietary treatment and aiming equal gender distribution and variety in litter. In addition, colon digesta samples were subjected to short-chain fatty acid (SCFA) analysis (n = 21 per dietary group).

### Culturing of bacteria

Culturing was performed as described in our previous study^8^. Digesta samples of 0.5 g from the ileum and colon of each pig were diluted in 4.5 ml 0.9% saline solution at collection, and subsequently serially diluted. Different selective agars (Oxoid, Cambridge, UK) were used for each bacterial group and 0.1 ml of the diluted digesta samples were plated. Lactic acid bacteria (LAB) were aerobically grown on de Man, Rogosa and Sharpe (MRS) agar at 35 °C for 48 h. *Enterococcus* spp. were grown on Slanetz and Bartley agar aerobically at 35 °C for 48 h. Coliforms were grown on MacConkey agar aerobically at 35 °C for 24 h. *C. perfringens* was grown on Tryptose Sulfite Cycloserine (TSC) agar anaerobically at 35 °C for 48 h. The following dilutions were used to plate the respective bacterial groups: 10^−6^, 10^−7^ and 10^−8^ for LAB; 10^−4^, 10^−5^ and 10^−6^ for *Enterococcus* spp.; 10^−3^, 10^−4^ and 10^−5^ for coliforms; 10^−3^, 10^−4^ and 10^−5^ for *C. perfringens*. The results were reported as log10 of colony-forming units (CFU) per gram digesta.

### DNA extraction and 16S *rRNA* gene sequencing

The DNA extraction and 16S rRNA gene sequencing were also performed as described in our previous study examining the effects of RSF on the gut microbiota of weaner piglets^8^, for comparability of the results from these two studies.

The digesta samples were processed for DNA extraction using QIAamp DNA Stool Mini Kit (Qiagen, GmbH, Hilden, Germany) protocol of the QIAcube system (Qiagen, GmbH, Hilden, Germany). The kit protocol was followed after a step of bead beating with zirconia/glass beads (diameter, 0.1 mm, Carl Roth, Karlsruhe, Germany) at 30 Hz for 2 min using the TissueLyser system (Qiagen Retsch GmbH, Hannover, Germany).

Quantification of the extracted DNA was performed by Qubit 3.0 Fluorometer using dsDNA Broad Range Assay Kit (Invitrogen, Eugene, OR, USA) and sent to GATC Biotech AG (Konstanz, Germany) for library preparation and sequencing of the V1-V3 variable region of 16S *rRNA* gene on Illumina MiSeq machine (Illumina, San Diego, CA, USA). This region was amplified using 27 F (AGAGTTTGATCCTGGCTCAG) and 534 R (ATTACCGCGGCTGCTGG) primers with output of 2 × 300 base-pair paired-end reads.

The raw sequencing files (fastq) have been deposited in the NCBI Sequence Read Archive (SRA) database (BioProject ID: PRJNA591752).

### Analysis of 16S *rRNA* gene sequencing data

The UPARSE pipeline^9^ implemented in USEARCH^10^ (version 9.2.64) was used to process the paired-end, de-multiplexed sequencing reads. The paired-end reads were merged, and quality filtering was applied using maximum expected error (maxee) value of 1. Sequences were dereplicated, and singletons were discarded. Operational taxonomic unit (OTU) clustering of sequences was performed using 97% sequence identity threshold, chimeric sequences were filtered from clustered OTUs using UCHIME^11^ and an OTU table was created. The further processing of OTUs were carried out using the Quantitative Insights Into Microbial Ecology (QIIME) version 1.9.1^12^. The representative OTUs were picked and aligned against the Greengenes core set database^13^ using PyNAST^14^ with the default minimum identity of 75%. The Ribosomal Database Project (RDP) classifier program^15^ was used to assign taxonomies to the aligned sequences at a confidence of 0.8. The OTUs with a fraction of total sequence count lower than 0.01 were filtered out from the OTU table. The OTU table was rarefied at the depth of 232963 sequences per sample for normalization. FastTree^16^ was used to build a phylogenetic tree from the aligned sequences after the filtration step to remove highly variable regions and positions that were all gaps. The phylogenetic tree was used to calculate alpha and beta diversity metrices.

### Imputed function prediction from 16S *rRNA* gene sequencing data

The functions of microbiome of CON- and RSF-fed pigs were predicted using PICRUSt^17^ online Galaxy version. A closed reference OTU table was generated from the filtered reads obtained from the sequencing analysis, using Greengenes core set database (May 2013 version) in QIIME^12^ v1.9.1. The steps were followed in the pipeline of PICRUSt online Galaxy version. Mainly, the generated closed reference OTU table was normalized by 16S rDNA copy number and metagenome was predicted. The predicted metagenomes were categorized by function based on Encyclopedia of Genes and Genomes (KEGG) pathways. The obtained file was processed by the linear discrimination analysis (LDA) effect size (LEfSe) algorithm^18^ online Galaxy version to characterize the significant differences between the feed groups. Default statistical parameters of α = 0.05 and LDA score 2.0 were used.

### Short-chain fatty acid (SCFA) analysis

The colon digesta samples were thawed on ice. A mixture of 500 mg of digesta sample and 500 µl of ice cold MQ-H_2_O was sonicated for 5 min in cold water. After mixing and centrifugation (15 min, 4 °C, 15000 g), the supernatant was transferred to a spin column (45 kDa). After another centrifugation step (15 min, 4 °C, 15000 g), supernatants were spiked with internal standard (2-methylvaleric acid). SCFA were measured by “TRACE 1300 Gas Chromatograph” equipped with a flame ionization detector and autosampler “AS 1310” (Thermo Fischer Scientific, Milan, Italy). The capillary column (Stabilwax-DA; 30-m × 0.25-mm i.d., 0,25 µm; Restek, Bellefonte, PA, USA) were operated as follows: starting temperature 90 °C (2 min); temperature increase 10 °C/min until 150 °C, 50 °C/min until 250 °C (1 min). The rate of helium flow was 3 mL/min. Concentrations of acetic, propionic acids, as well as butyric, valeric acids and their isomers were quantified against external standards using Chromeleon software (Dionex, Thermo Scientific) and reported in µmol per gram of intestinal contents.

### Statistical analysis

The statistical analyses of microbiota data and the correlation analyses were performed using the JMP Pro software (version 13, SAS Institute Inc.). Normality of the data was tested using the Shapiro-Wilk test^19^. Based on the normality of the data, culturing data were compared between the dietary treatments using student’s t-test corrected with false discovery rate (FDR); while the alpha diversity indices were analyzed using the non-parametric Kruskal–Wallis test with Dunn’s multiple comparison post-hoc test. The OTU table was filtered prior to the differential abundance analysis for the bacterial taxa, such that only the OTUs that were observed at least 100 times among all the samples and in at least 15 samples were kept for the analysis. The differentially abundant taxa were characterized using the linear discrimination analysis effect size (LEfSe) algorithm online Galaxy version, with parameters of α = 0.05 and LDA score 2.0. Principle coordinate analysis (PCoA) and permutational multivariate analysis of variance (PERMANOVA) on the unweighted and weighted UniFrac distance metrics were performed using the web-based tool, MicrobiomeAnalyst^20^.

## Results

### Culturing of coliforms, *Enterococcus* spp., lactic acid bacteria (LAB) and *C. perfringens*

**Figure 1:**
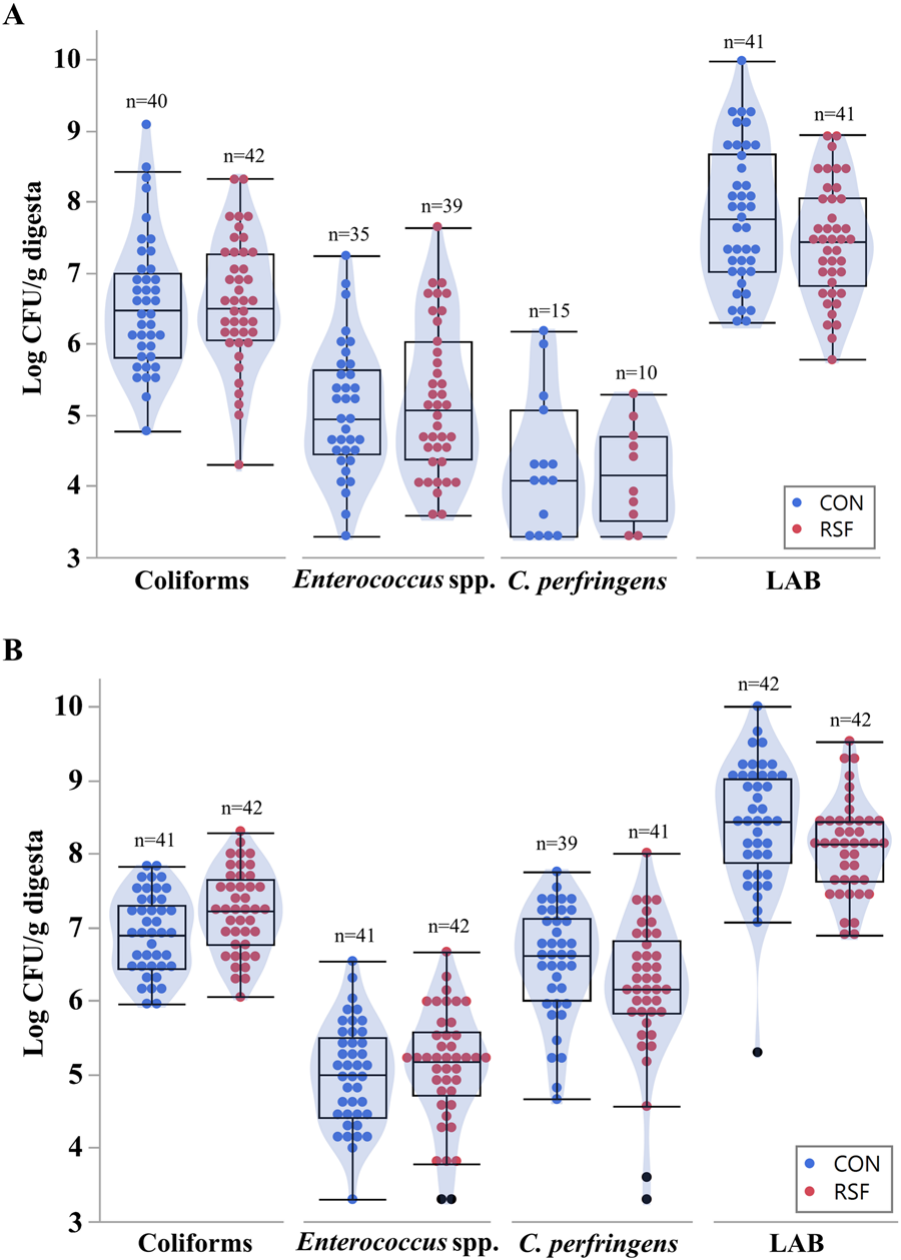
The counts of cultured bacterial groups in the ileum and colon of pigs fed with CON and RSF. (**A**) Ileum, (**B**): Colon. LAB: Lactic acid bacteria. The number of samples included in the analysis (n) is indicated for every treatment and bacterial group on the plots.

The coliforms, *Enterococcus* spp., LAB and *C. perfringens* were cultured only for the ileum and colon digesta samples. None of these bacterial groups were differentially abundant between the CON and RSF groups in the ileum (Fig. 1A). In colon of RSF pigs, LAB and *C. perfringens* populations tended to be less abundant (*P-value* = 0.065) compared with CON pigs (Fig. 1B).

### Gut microbiome diversity and composition in the ileum, caecum and colon of CON- and RSF-fed pigs

For the comparison of alpha and beta diversities in CON- and RSF-fed pigs, sequencing of the V1-V3 region of 16S *rRNA* gene extracted from 3 gut location of 48 pigs (144 samples in total) was performed. The sequencing resulted in 70053289 filtered, high-quality sequences with a mean of 486481 sequences per sample and these sequences were clustered into 2165 OTUs at 97% identity threshold. Alpha diversity indices, i.e. Shannon index, observed OTUs, Chao1 and Phylogenetic diversity whole tree, were calculated for the treatment groups in the ileum, caecum and colon (Table 2). Despite of the differences between the gut locations as expected due to the increasing bacterial load from the small intestine to the large intestine, diversity was similar between the diet groups within each location.

**Table 2:** Comparison of alpha diversity indices at OTU level in the ileum, caecum and colon of CON- and RSF-fed pigs.

Group	Mean values^a^			
	Shannon	Observed OTUs	Chao1	Phylogenetic diversity whole tree
i_CON	3.4^C^	550^D^	709^C^	26^C^
i_RSF	3.7^C^	557^D^	733^C^	27^C^
ca_CON	6.7^AB^	1201^BC^	1345^B^	44^B^
ca_RSF	6.4^B^	1112^C^	1260^B^	43^B^
co_CON	7.2^A^	1407^A^	1510^A^	51^A^
co_RSF	6.9^AB^	1303^AB^	1407^AB^	49^A^
**Pooled SEM**	0.14	32	31	0.95

^a^Different letters within the same column indicate significant difference (p < 0.05)

i_CON, ca_CON and co_CON: ileum, caecum and colon of CON-fed pigs respectively; i_RSF, ca_RSF and co_RSF: ileum, caecum and colon of RSF-fed pigs respectively.

**Figure 2:**
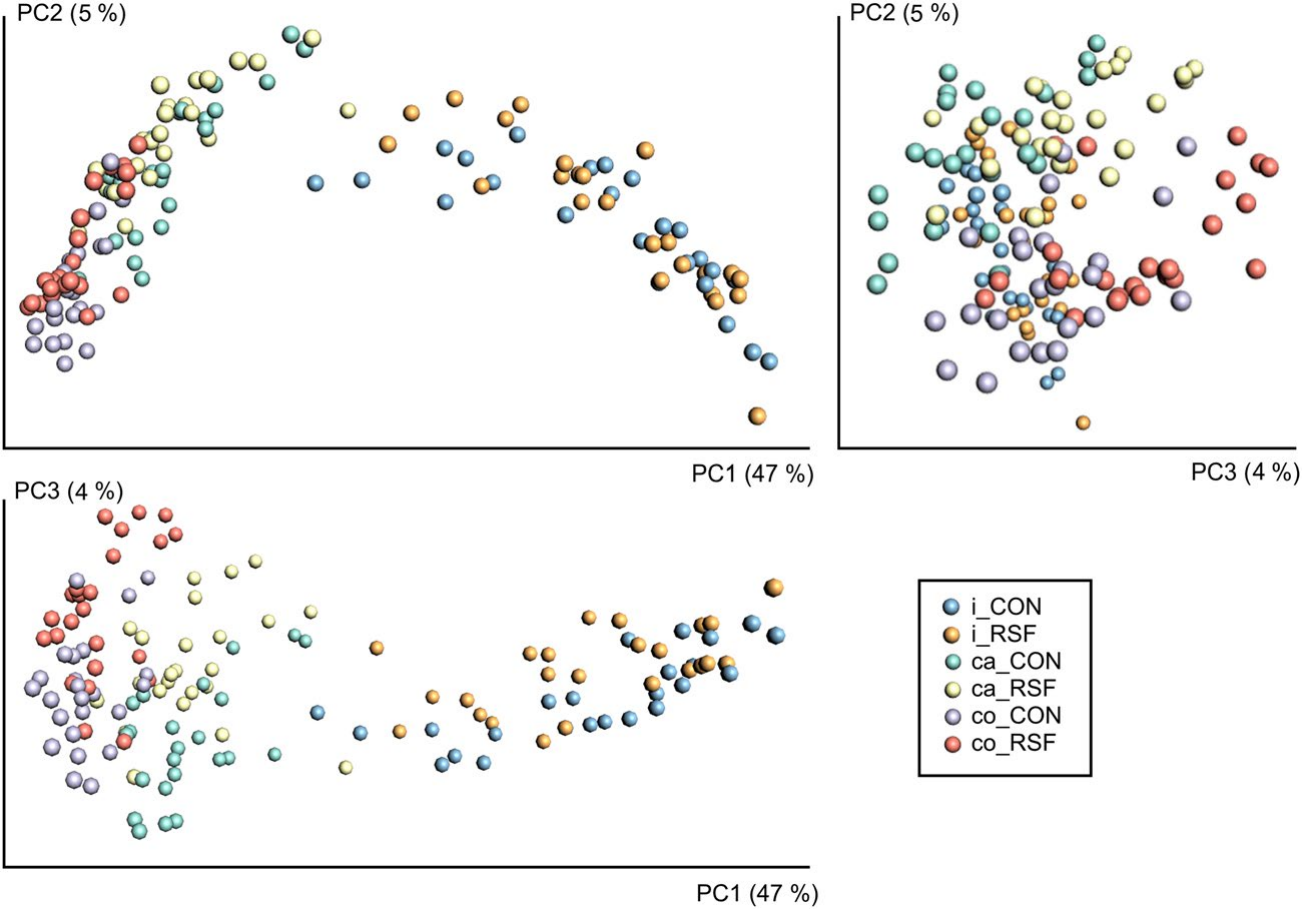
Bacterial compositions in the ileum, caecum and colon of CON- and RSF-fed pigs. Principle coordinate analysis (PCoA) plots were generated based on the calculated distances in the unweighted UniFrac matrix. Samples were grouped by color based on the gut location and feed type. i, ileum; ca, caecum; co, colon.

The beta diversity was analyzed using phylogenetic unweighted and weighted Unifrac dissimilarity measures, and the matrices were visualized using Principle Coordinate Analysis (PCoA). Unweighted UniFrac metric results are presented in Fig. 2 and weighted UniFrac metric results are available in Supplementary Fig. S1. Overall, microbiome composition differed between the gut locations regardless of the dietary treatment, with a more remarkable divergence of the ileum samples. PCoA generated based on unweighted UniFrac metric, showed that 47% of the variation in the gut microbiome composition could be explained by the first axis (PC1) and clearly separated the gut locations (Fig. 2). The weighted UniFrac metric analysis supported this, explaining 86% of the variation in composition and the ileum samples being more pronounced. The variations in microbiome composition mainly of caecum and colon between the CON- and RSF-fed pigs (PERMANOVA, *P-value* = 0.001 for the caecum and colon, and *P-value* > 0.05 for the ileum) were explained by the third axis (PC3), but the distinguished variation was only 4% based on the unweighted UniFrac metric (Fig. 2) and 1.8% based on the weighted UniFrac metric (Supplementary Fig. S1).

Overall, the ileum samples were dominated by Firmicutes phylum, while Bacteroidetes was the most and Firmicutes the second most abundant phyla in caecum and colon samples in both CON and RSF pigs. The other phyla detected in the samples and the relative abundances can be found in Supplementary Table S1. The relative abundances of bacteria at phylum level were not affected by the RSF feeding in any of the gut locations.

### Effect of RSF on the relative abundance of bacterial phylotypes

**Figure 3:**
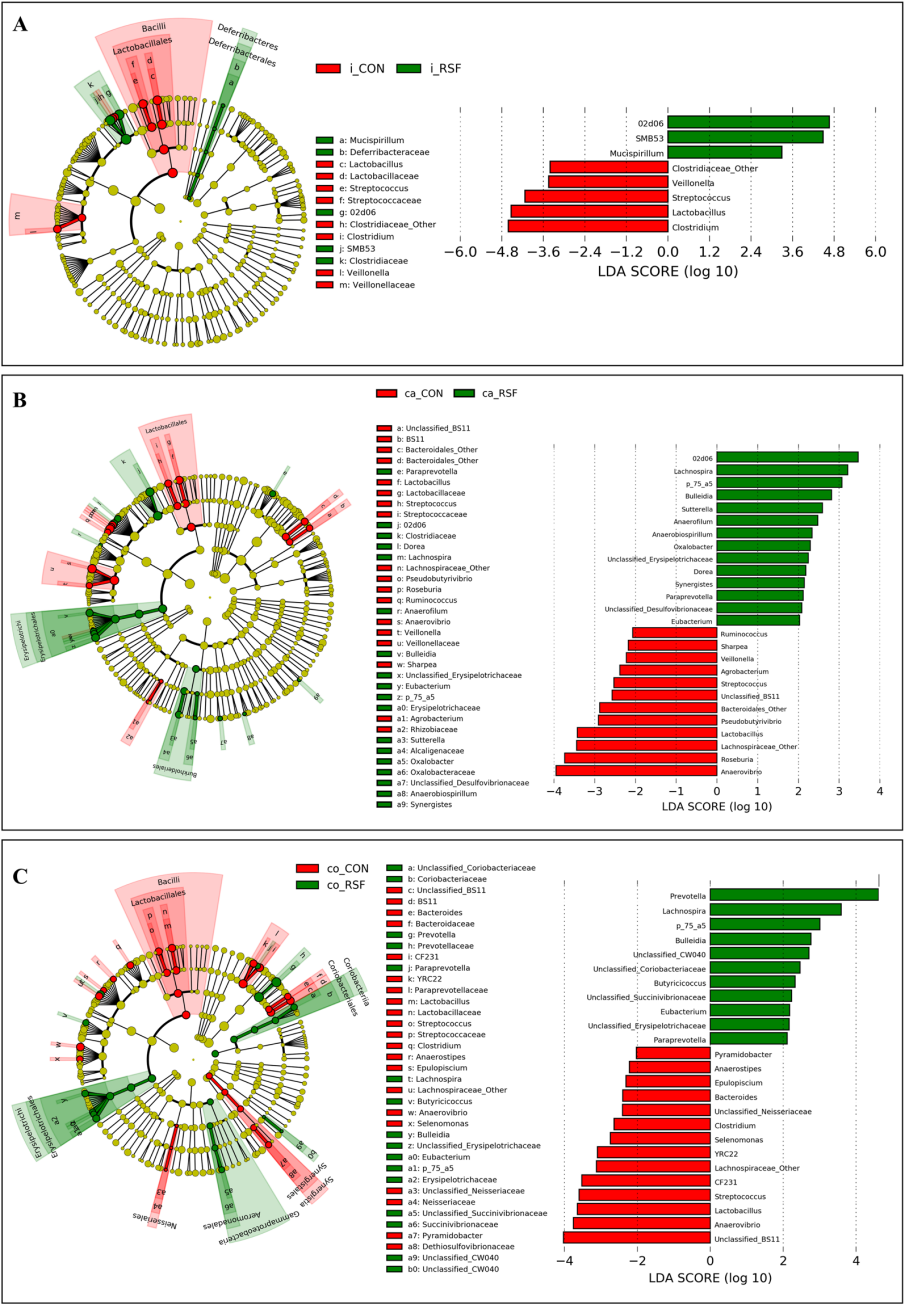
Discriminative bacterial taxa between the CON and RSF groups based on the relative abundance data. (**A**), ileum; (**B**), caecum and (**C**), colon.

Differential abundance analysis was performed for the bacterial taxa in the ileum, caecum and colon of the CON- and RSF-fed pigs. Several phylotypes were found to be more abundant in one of the dietary treatment groups in the ileum, caecum and colon of the pigs (Fig. 3).

In the ileum of RSF-fed pigs (Fig. 3A), Clostridiaceae family and *02d06* and *SMB53* genera affiliated to this family, were more abundant. In addition, *Mucispirillum* genus, the only genus affiliated to Deferribacteres phylum found in the samples, was more abundant in the RSF group ileum samples. On the other hand, Bacilli class of bacteria, particularly *Lactobacillus* and *Streptococcus* from the Lactobacillales order, and the *Veillonella* genus from Veillonellaceae family were more predominant in the ileum samples from the CON-fed pigs.

In the large intestine, more phylotypes were differentially abundant between the CON and RSF treatments (Fig. 3B,C, Supplementary Table S1). In caecum, the differently abundant phylotypes between CON and RSF treatments followed a similar trend to the ileum, such that *02d06* genus affiliated to the Clostridiaceae family was more abundant in RSF group while *Veillonella* genus in CON group. As with the ileum, in both caecum and colon samples, the *Lactobacillus* and *Streptococcus* genera were more abundant in CON group. In both caecum and colon of RSF-fed pigs, Erysipelotrichaceae family members, namely *Bulleidia*, *Eubacterium*, *p_75_a5* genera and an unclassified genus (Unclassified_Erysipelotrichaceae), as well as *Lachnospira* and *Paraprevotella* were more abundant. Moreover, genera *Dorea*, *Anaerofilum*, *Sutterella* and *Oxalobacter* affiliated to Burkholderiales order, *Anaerobiospirillum*, *Synergistes* and an unclassified Desulfovibrionaceae genus were more abundant in caecum of RSF-fed pigs. The genera that were predominant in only colon of RSF-fed pigs included *Prevotella*, *Butyricicoccus* and an unclassified Succinivibrionaceae genus and an unclassified CW040 genus affiliated to TM7 phylum. On the other hand, CON group pigs had a higher abundance of an unclassified BS11 genus affiliated to Bacteroidales order and *Anaerovibrio* genus in both caecum and colon. Higher abundances of a genus affiliated to Bacteroidales (Bacteroidales_Other), *Pseudobutyrivibrio*, *Roseburia*, *Ruminococcus*, *Sharpea*, and *Agrobacterium* genera were observed in caecum of CON-fed pigs; and *CF231* and *YRC22* affiliated to Paraprevotellaceae, *Clostridium*, *Anaerostipes*, *Epulopiscium*, *Anaerovibrio*, *Selenomonas*, and unclassified Neisseriaceae genus and *Pyramidobacter* genera in colon of CON-fed pigs.

In both caecum and colon, the same genera, *Lactobacillus* and *Streptococcus* from Bacilli class were more abundant in CON-fed pigs, which was similar to the findings in the ileum.

### Functional diversity based on dietary treatments

**Figure 4:**
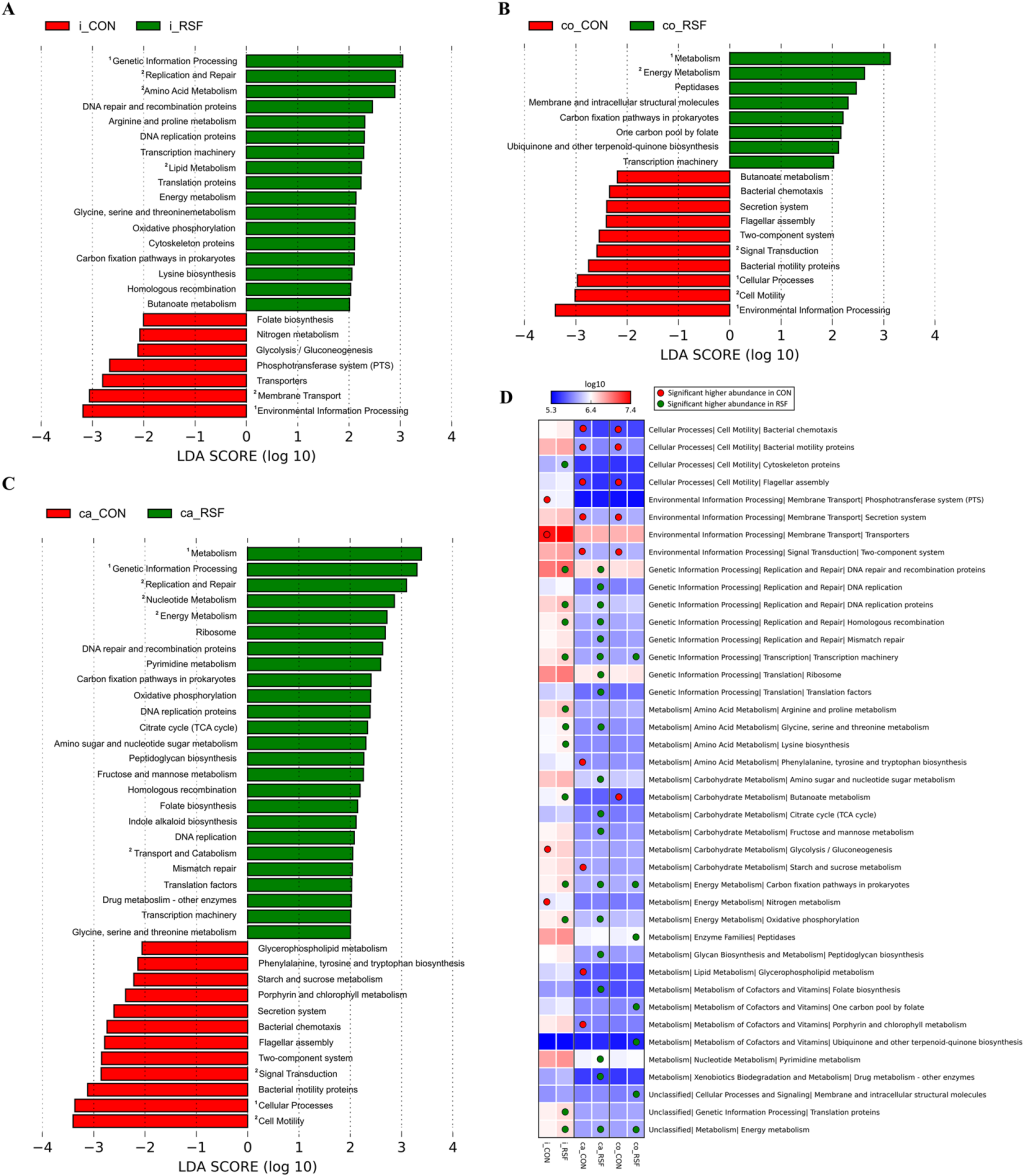
Predicted functional capacity profiles of the bacterial communities in the ileum (**A**), caecum (**C**) and colon (**B**) of the CON- and RSF-fed pigs. Microbial functions were predicted using PICRUSt at the third level of the KEGG pathway, “^1^” next to the function name indicates level 1 and “^2^” level 2 while other functions are at the third level. The heatmap (**D**) shows the mean gene abundances (log transformed) per treatment for each gut location, which were significantly affected by the dietary treatment in at least one of the gut locations as indicated by colored circles.

Microbiome functions of the ileum, caecum and colon of CON- and RSF-fed pigs were predicted from the 16S *rRNA* gene sequences to investigate whether the RSF feeding caused functional changes within the microbiome of pigs compared with CON feeding. The imputed relative abundances of KEGG pathways in each respective sample were compared between the CON and RSF groups to examine the differences in metabolic function. We observed differentially abundant KEGG pathways in all the gut locations analyzed (Fig. 4). In general, genetic information processing pathways, including replication and repair, transcription and translation pathways, and energy metabolism pathways, particularly carbon fixation pathways in prokaryotes, were found to be more abundant among the gut microbiome of RSF pigs. In addition, in the ileum, amino acid metabolism pathways such as arginine and proline metabolism, glycine, serine and threonine metabolism and lysine metabolism, and energy metabolism pathway - oxidative phosphorylation were more abundant in RSF pigs. In the caecum, similar to the ileum, abundance of glycine, serine and threonine metabolism function and energy metabolism pathway - oxidative phosphorylation were higher in RSF pigs. Additional functions enriched in caecum of RSF pigs included carbohydrate metabolism pathways, such as amino sugar and nucleotide sugar metabolism, citrate cycle (TCA cycle) and fructose and mannose metabolism, and peptidoglycan biosynthesis, folate biosynthesis and pyrimidine metabolism. In colon of RSF pigs, in addition to the aforementioned functions, higher abundance of metabolism of cofactors and vitamins, namely one-carbon pool by folate and ubiquinone and other terpenoid-quinone biosynthesis compared with the colon of CON pigs was found.

On the other hand, environmental information processing pathways, phosphotransferase system (PTS) and transporters in the ileum and secretion system and two-component system in both caecum and colon were less abundant in RSF pigs. Moreover, a lipid metabolism pathway, glycerophospholipid metabolism was less abundant in caecum, while a carbohydrate metabolism pathway, butanoate metabolism was less abundant in colon of RSF pigs.

## Discussion

Previously, we have investigated the effect of feeding a sustainable high-fiber rapeseed meal-based diet on the gut microbiota of weaner pigs and reported the potential of this diet to modulate the gut microbiota in a beneficial way for the weaner piglets^8^. The aim of this study was to extend our findings on the health-related consequences of use of rapeseed meal as an alternative protein source to soybean meal in the pig industry. Therefore, we examined how the gut microbiota of growing-finishing pigs respond to RSF after a three-month feeding treatment compared with CON. According to the meta-analysis we conducted recently^21^, the rapeseed meal shows no adverse effects on growth performance of growing-finishing pigs when used as protein source in nutritionally balanced diets. In our trial, RSF reduced growth performance, carcass weight and dressing percentage in pigs compared to CON, but had no effect on the meat quality traits^2^.

The effects of RSF on bacterial communities of the ileum, caecum and colon of growing-finishing pigs were investigated using both the culturing and 16S *rRNA* gene sequencing approaches. The results from culturing of certain bacterial groups correlated with the sequencing results, confirming reliability of our results. Based on culturing, LAB counts tended to be lower in colon of RSF-fed pigs compared with CON pigs, concordantly *Lactobacillus* genus was less abundant in RSF pig gut based on sequencing data results. It has been shown that *Lactobacillus* predominates in the gut of pigs at early stage of life and its abundance shows a strong negative association with age, almost disappearing after the growing period^22,23^. Therefore, its presence, reputable probiotic activity and beneficial functions are most likely more influential in young pigs, and our previous study on weaner piglets has not shown any reduction of *Lactobacillus* in gut of the RSF-fed pigs^8^. Moreover, despite the decreased abundance of this beneficial bacterial group in RSF pigs, the imputed function of the microbiome was not adversely affected in RSF group (discussed further below). These findings support the notion that it is the overall function of the whole microbial community that is the determinative factor for gut health of the host.

The RSF had a similar effect on the relative abundance of bacterial phylotypes in gut of growing-finishing pigs compared with those in gut of weaner piglets, although the diets were slightly different with an additional 4% pure rapeseed hulls in the previous study^8^. On the other hand, we have observed that the response of bacteria in the ileum was more pronounced in growing-finishing pigs, most probably due to possessing relatively well-established and mature microbiota compared with weaner piglets. Interestingly, the *Mucispirillum* genus affiliated to the Deferribacteres phylum was more predominant in the ileum of RSF-fed pigs. This group of bacteria has been shown to colonize the mucus layer and closely interact with the intestinal epithelium in ileum in mice and human^24,25^. It has been included in the minority of gut microbes that trigger a T-dependent immunoglobulin A (IgA) response and are critical for immunity induction and regulation^26^. Moreover, recently it has been reported that a species affiliated to this genus, *Mucisprillium schaedleri*, antagonizes *Salmonella* virulence and restricts infection in the gut of mice to protect the host against colitis^27^. Therefore, the predominance of this bacterial population in RSF group is interesting and merits further immunological work to confirm the similar effects in pigs.

It is known that the gut microbiota has functional redundancy such that despite compositional differences in the members, the community behaviour may be similar^28^, and we have previously observed such a functional redundancy in weaner pigs when fed with RSF^8^. Therefore, we have analyzed and compared the imputed functions of the gut microbiome between the CON and RSF groups to see whether the gut microbiota of growing-finishing pigs responds similarly when fed with RSF despite the differentially abundant phylotypes found. Differences were observed in the abundance of some imputed functions of the ileal, cecal and colonic microbiota when the growing-finishing pigs were fed with the RSF. The modulation of the imputed functions of microbiome occurred gradually from the ileum to colon, with caecum having some common changes observed in the ileum or colon. Overall, genetic information processing pathways, including replication and repair, transcription and translation pathways were more abundant among the gut microbiome of RSF pigs. This response of the microbiota indicates higher microbial proliferation and activity in the gut of RSF pigs and is in accordance with the higher content of both neutral and acid detergent fibers of RSF^2^, which act as a substrate for the bacteria. These findings were also consistent with the enhancement of several energy and carbon metabolism pathways in microbiome of RSF group, which are characteristic of the anaerobic bacteria that can degrade the plant cell wall components in the diet and affiliated to SCFA production^29^. RSF feeding resulted in predominance of putative fiber fermenting phylotypes, such as members of Prevotellaceae, Erysipelotrichaceae, Clostridiaceae, *Lachnospira*, *Butyricicoccus* in the large intestine although we have not seen any increase in measured SCFA levels in colon of RSF pigs (Supplementary Information SI). Most SCFAs are very quickly absorbed from the intestine, thus the concentrations of luminal SCFA do not always reflect the total production of SCFA^30^. Moreover, in our study, the pigs were fed 2–2.5 hours before sampling to ensure enough content in the small intestine, thus there may not be full production of SCFA in the hindgut at the sampling time point. The predicted enrichment of the citrate cycle (TCA cycle) pathway supports enhanced fermentative activity and bacterial energy acquisition in RSF pigs. Enrichment of this function, which is associated with lower available oxygen, indicates contribution of the RSF to improvement of anaerobic environment in gut, which is less favorable for pathogen growth^31^. For instance, the higher abundance of Succinivibrionaceae populations in colon and particularly the *Anaerobiospirillum* genus in caecum of RSF pigs supports this, since this group of bacteria is strictly anaerobe, thrives in the presence of carbon dioxide and ferments carbohydrates to produce acetate and succinate^32^.

Furthermore, the metabolic pathways providing intermediates into the TCA cycle pathway seemed to vary between the diets in caecum. In CON pigs, starch and sucrose metabolism probably provided the necessary precursors via the glycolysis pathway^33^. On the other hand, the metabolites from the fructose and mannose metabolism pathway may have played the major role as the precursors for TCA cycle, also for amino sugar and nucleotide sugar metabolism^33^ in the caecum of RSF pigs. Thereby, stimulating the biosynthesis of vitamin B^34^, as we have found folate biosynthesis pathway to be more predominant in the caecum of RSF-fed pigs. Mammals need vitamins supplemented in their diet or rely on vitamin synthesis by gut commensal bacteria since mammals cannot synthesize the vitamins that they require. Furthermore, although the absorption of vitamins is less efficient in large intestines, it can still be utilized by pigs^35^.

In addition to the enrichment of the folate biosynthesis pathway in caecum by RSF, one-carbon pool by folate pathway, which uses this vitamin as a cofactor, was found to be higher in colon of RSF pigs. Folate is used as a cofactor in several important metabolic reactions, such as DNA synthesis (biosynthesis of purines and thymidine) and amino acid homeostasis (glycine, serine, and methionine)^36,37^. Moreover, folate supports the maintenance of immunological homeostasis, as it is a survival factor for regulatory T (Treg) cells and crucial for their maintenance^37,38^. In addition to immunological effects, folate intake is particularly important for sows, as it has been shown to improve the reproductive performance of sow by increasing the number of piglets born alive^39^ and by increasing the milk production and quality of lactating sows and the growth performance of piglets^40^. The use of high fiber ingredients in the diet of sows has previously been suggested to be favorable for their welfare and reproductive performance^41^. Therefore, the modulation of the microbiota by RSF feeding is promising to improve both the immunological homeostasis and performance of sows and suckling pigs. It should be emphasized that these are imputed functions of microbiota related to folate and these *in silico* findings need to be supported by further studies.

In parallel to the one-carbon metabolism pathway enrichment, glycine, serine and threonine metabolism was more abundant in the RSF pigs. Particularly, glycine and serine are important intermediate metabolites in one-carbon metabolism and have been previously shown to be increased and decreased, respectively, by rapeseed-based feed in liver of weaner pigs^42^. It has been questioned whether the change in levels of these amino acids may be due to the rapeseed-based feed affecting the expression levels of the serine hydroxymethyltransferase enzyme. However, transcriptomics analysis of liver samples from some pigs in the present experiment has shown no change in expression of serine hydroxymethyltransferase enzyme-related genes (data not shown). Therefore, our findings suggest that the RSF effect on the gut microbiota has an impact on the conversion of serine into glycine, possibly affecting the levels of these amino acids in the liver of pigs.

The butanoate metabolism was predicted to be more predominant in the ileum of RSF pigs, and on the contrary less in colon of RSF group. On the other hand, no significant difference was found in butyric acid levels in colon (not measured in ileum) between the dietary treatment groups. It is known that inorganic ions, such as nitrate and sulfate, profoundly affect the carbohydrate fermentation reactions under anaerobic conditions, typically resulting in reduced butyrate and lactate production^33^. The RSF in our study contains a relatively high amount of glucosinolates that include nitriles and sulfates^43^, which is different from the CON. Therefore, this component may be responsible for the shifts in fermentation reactions and regulation of short chain fatty acid production resulting in reduced butyrate metabolism capacity of the microbiome in large intestine of the RSF-fed pigs compared with the CON group. The effect of the sulfate content in the RSF was seen with the enhancement of sulfate-reducing bacterial groups in caecum of RSF-fed pigs, e.g. an unclassified Desulfovibrionaceae genus^44^. The opposing trend found in butanoate metabolism in the ileum and colon is interesting since butyrate regulation has been suggested to play different roles in ileum and colon, providing an immune induction in ileum but immune tolerance in colon^26^. Taken together with other effects of RSF, such as predominance of the immune-inducing bacterial group *Mucispirillum* in the ileum, anti-inflammatory stimulating bacteria *Lachnospira* and *Coprococcus*^8^ in the large intestine, and promoting robustness of the gut microbiota in case of post-weaning diarrhea^8^, the RSF seems to play a role in induction of the immune system via modulation of the gut microbiota in favor of the host.

The feeding of RSF to weaner pigs has previously shown a reduced apparent ileal digestibility of amino acids due to the high fiber content of rapeseed by-products^3^. In accordance with these observations, we have found that the putative functions related to amino acid metabolism were enriched in the ileum of RSF since the apparent ileal digestibility of amino acids were lower in the RSF pigs than in the CON pigs and amino acids were thus available for the metabolism of microbiota. On the other hand, our results suggest that higher fiber content of RSF stimulated the metabolism of carbohydrate instead of protein fermentation that negatively affects both the gut health and the environment^45^.This may have led to increased fiber degraders at the expense of protein degraders. This outcome can explain the higher abundance of major amino acid fermenters and amylolytic bacteria^46^ in gut of CON pigs compared with RSF pigs. These bacterial populations included *Streptococcus* in all the ileum, caecum and colon, *Veillonella* in the ileum and caecum, *Clostridium* in both the ileum and colon, and *Bacteroides* and *Selenomonas* genera in colon. Another amino acid metabolism pathway affected by RSF was lysine biosynthesis pathway. It was enhanced in the ileum of RSF pigs. Lysine is an essential amino acid for pigs and the absorption of lysine, produced by the microbiota, occurs mostly in small intestine^47^.

Compared with the RSF group, the large intestinal gut microbiome of the CON group possessed a reduced potential for carbohydrate and energy metabolism and an enhanced potential for some cell motility pathways, namely bacterial chemotaxis, bacterial motility proteins and flagellar assembly, as well as secretion system and two-component system pathways that were categorized under environment information processing. The mentioned cell motility functions, abundant in the caecum and colon of the CON pigs, have been associated with bacterial pathogenicity and implicated as disease drivers in several animal models^48–50^. Secretion systems have been evolved by pathogens and used to survive in, colonize and infect the host^51^. Two-component systems are signaling pathways that control basic cellular processes of bacteria such as nutrient uptake, secondary metabolite production and cell division and enable adaptation to a changing environment; on the other hand, they are used by pathogenic bacteria to control virulence^51^. In the light of these findings, if the genes associated with these functions are truly expressed, the gut microbiome of the RSF-fed pigs seems to be less prone to possible infection or inflammation caused by opportunistic pathogens compared with CON-fed pigs.

## Conclusion

Our findings support the favorable effect of rapeseed meal on the gut microbiome, and indicate a potential for the protection of pigs from infections and improved immunological homeostasis via modulation of gut microbiome functions. We have found some results that may seem undesirable, such as reduction in *Lactobacillus* abundance; however, the functional prediction of the microbiome shows that bacterial community interactions in the gut is very complex and the overall functions of the microbiome as a community outweighs the contribution of a single member of that community. It is the functions of the microbiome that affects the host physiology and health. Therefore, enhancement of the gut microbiota imputed health functions, the sustainable production of rapeseed with less environmental impact and the lower cost of rapeseed by-products overall argue that rapeseed by-products could be a good local alternative to imported soybean meal.

## Supplementary information

Supplementary Information.

Supplementary Information 2.

Supplementary Information 3.
