# Supplementary material for: Rapeseed-based diet modulates the imputed functions of gut microbiome in growing-finishing pigs
Source: Sci Rep. 2020 Jun 10;10:9372. doi: 10.1038/s41598-020-66364-4 (PMC7287078; doi:10.1038/s41598-020-66364-4)
Supplement: Supplementary file 1 — Supplementary Information. [file 41598_2020_66364_MOESM1_ESM.docx]

**Rapeseed-based diet modulates the imputed functions of gut microbiome in growing-finishing pigs**

Özgün Candan Onarman Umu^1*^: ozgun.umu@nmbu.no

Liv Torunn Mydland^2^: liv.mydland@nmbu.no

Margareth Øverland^2^: margareth.overland@nmbu.no

Charles McLean Press^3^: charles.press@nmbu.no

Henning Sørum^1^: henning.sorum@nmbu.no

^1^Department of Paraclinical Sciences, Faculty of Veterinary Medicine, Norwegian University of Life Sciences, Oslo, Norway.

^2^Department of Animal and Aquacultural Sciences, Faculty of Biosciences, Norwegian University of Life Sciences, Ås, Norway

^3^Department of Preclinical Sciences and Pathology, Faculty of Veterinary Medicine, Norwegian University of Life Sciences, Oslo, Norway

*Corresponding author


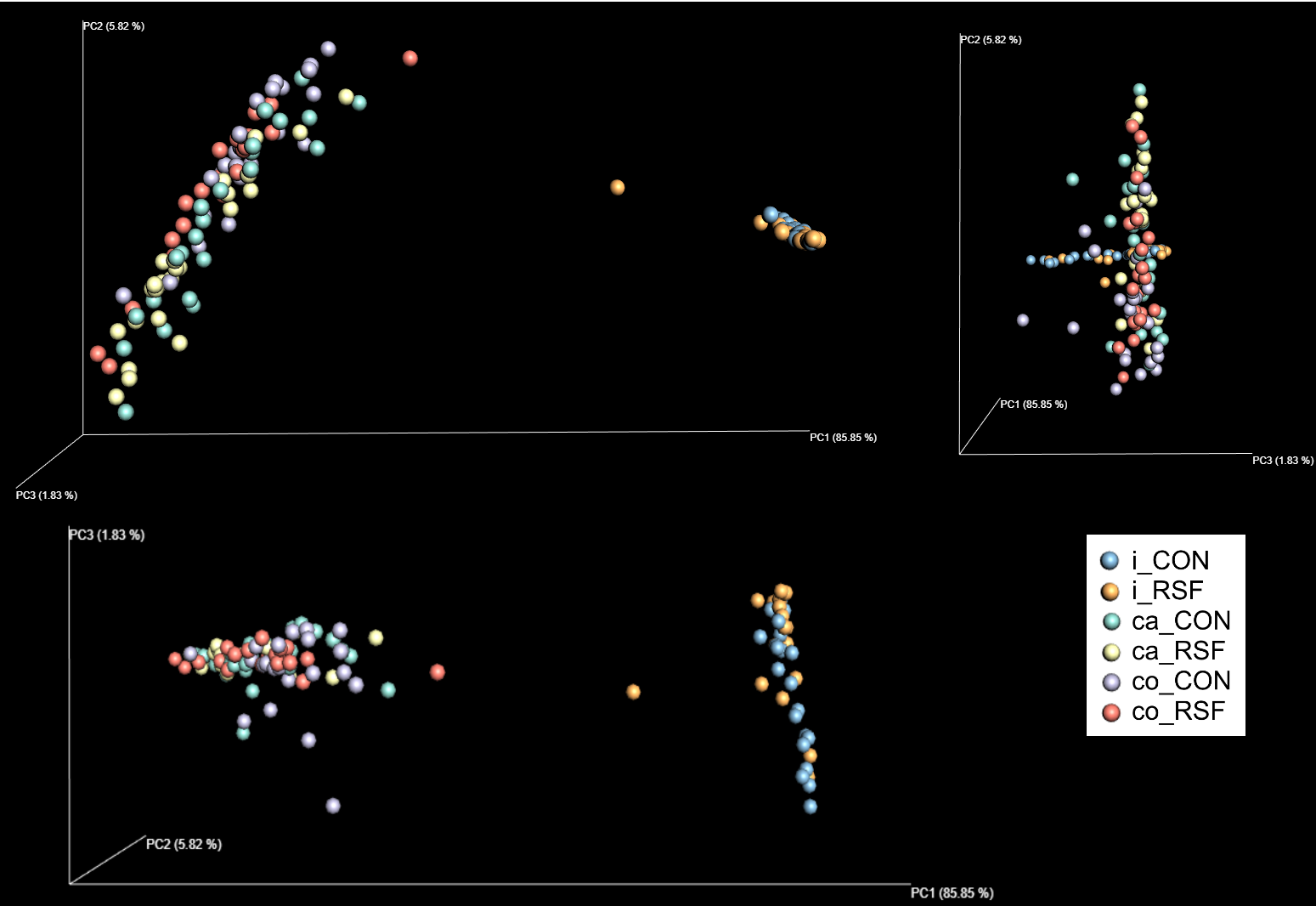


**Figure S1.** Bacterial compositions in ileum, caecum and colon of CON- and RSF-fed pigs based on the calculated distances in the weighted UniFrac matrix. Samples were grouped by color based on the gut location and feed type. i, ileum; ca, caecum; co, colon.
