# Supplementary material for: Rapeseed-based diet modulates the imputed functions of gut microbiome in growing-finishing pigs
Source: Sci Rep. 2020 Jun 10;10:9372. doi: 10.1038/s41598-020-66364-4 (PMC7287078; doi:10.1038/s41598-020-66364-4)
Supplement: Supplementary file 2 — Supplementary Information 2. [file 41598_2020_66364_MOESM2_ESM.doc]

**Rapeseed-based diet modulates the imputed functions of gut microbiome in growing-finishing pigs**

Özgün Candan Onarman Umu^1*^: ozgun.umu@nmbu.no

Liv Torunn Mydland^2^: liv.mydland@nmbu.no

Margareth Øverland^2^: margareth.overland@nmbu.no

Charles McLean Press^3^: charles.press@nmbu.no

Henning Sørum^1^: henning.sorum@nmbu.no

^1^Department of Paraclinical Sciences, Faculty of Veterinary Medicine, Norwegian University of Life Sciences, Oslo, Norway.

^2^Department of Animal and Aquacultural Sciences, Faculty of Biosciences, Norwegian University of Life Sciences, Ås, Norway

^3^Department of Preclinical Sciences and Pathology, Faculty of Veterinary Medicine, Norwegian University of Life Sciences, Oslo, Norway

*Corresponding author

**Supplementary Information (SI):**

**The short-chain fatty acid (SCFA) analysis statistics and results:**

The SCFA analysis has been performed as described in the “Materials and Methods” section of the main text. Based on the statistical analysis shown below, there is no significant difference in any of the SCFAs measured between the CON and RSF treatments.

**Fit Group**

**Oneway Analysis of Acetic acid By Treatment**

**Quantiles**

| **Level** | **Minimum** | **10%** | **25%** | **Median** | **75%** | **90%** | **Maximum** |
| --- | --- | --- | --- | --- | --- | --- | --- |
| CON | 35,09528 | 42,78883 | 46,21129 | 56,72221 | 64,03836 | 69,14948 | 69,97931 |
| RSF | 32,93647 | 38,96123 | 46,84088 | 54,25371 | 55,80956 | 61,52353 | 80,1921 |

**t Test**

RSF-CON

Assuming unequal variances

| Difference | -2,7666 | t Ratio | -0,90672 |
| --- | --- | --- | --- |
| Std Err Dif | 3,0512 | DF | 39,93824 |
| Upper CL Dif | 3,4004 | Prob > |t| | 0,3700 |
| Lower CL Dif | -8,9336 | Prob > t | 0,8150 |
| Confidence | 0,95 | Prob < t | 0,1850 |

**Oneway Analysis of Propionic acid By Treatment**

**Quantiles**

| **Level** | **Minimum** | **10%** | **25%** | **Median** | **75%** | **90%** | **Maximum** |
| --- | --- | --- | --- | --- | --- | --- | --- |
| CON | 15,81157 | 19,38188 | 20,70838 | 26,1544 | 31,31516 | 45,65444 | 49,15212 |
| RSF | 16,23559 | 17,5496 | 19,92264 | 25,53135 | 30,36565 | 34,73073 | 36,05862 |

**t Test**

RSF-CON

Assuming unequal variances

| Difference | -2,0907 | t Ratio | -0,88734 |
| --- | --- | --- | --- |
| Std Err Dif | 2,3562 | DF | 34,32587 |
| Upper CL Dif | 2,6959 | Prob > |t| | 0,3811 |
| Lower CL Dif | -6,8773 | Prob > t | 0,8095 |
| Confidence | 0,95 | Prob < t | 0,1905 |

**Oneway Analysis of Iso butyric acid By Treatment**

**Quantiles**

| **Level** | **Minimum** | **10%** | **25%** | **Median** | **75%** | **90%** | **Maximum** |
| --- | --- | --- | --- | --- | --- | --- | --- |
| CON | 0,448964 | 0,734188 | 1,080681 | 1,618448 | 1,864702 | 2,428026 | 2,899987 |
| RSF | 0,165231 | 0,753418 | 1,038842 | 1,3884 | 1,849336 | 2,325255 | 2,38187 |

**t Test**

RSF-CON

Assuming unequal variances

| Difference | -0,16198 | t Ratio | -0,91613 |
| --- | --- | --- | --- |
| Std Err Dif | 0,17681 | DF | 39,95271 |
| Upper CL Dif | 0,19538 | Prob > |t| | 0,3651 |
| Lower CL Dif | -0,51934 | Prob > t | 0,8175 |
| Confidence | 0,95 | Prob < t | 0,1825 |

**Oneway Analysis of Butyric acid By Treatment**

**Quantiles**

| **Level** | **Minimum** | **10%** | **25%** | **Median** | **75%** | **90%** | **Maximum** |
| --- | --- | --- | --- | --- | --- | --- | --- |
| CON | 7,404814 | 10,36787 | 12,19575 | 14,59982 | 17,86665 | 19,98028 | 21,0715 |
| RSF | 4,869886 | 9,362154 | 11,59589 | 14,51481 | 16,01884 | 18,92776 | 22,14816 |

**t Test**

RSF-CON

Assuming unequal variances

| Difference | -0,7913 | t Ratio | -0,70233 |
| --- | --- | --- | --- |
| Std Err Dif | 1,1267 | DF | 39,67919 |
| Upper CL Dif | 1,4864 | Prob > |t| | 0,4866 |
| Lower CL Dif | -3,0690 | Prob > t | 0,7567 |
| Confidence | 0,95 | Prob < t | 0,2433 |

**Oneway Analysis of Iso valeric acid By Treatment**

**Quantiles**

| **Level** | **Minimum** | **10%** | **25%** | **Median** | **75%** | **90%** | **Maximum** |
| --- | --- | --- | --- | --- | --- | --- | --- |
| CON | 0,36046 | 0,640534 | 0,889696 | 1,704942 | 2,133948 | 2,820619 | 4,000913 |
| RSF | 0,178481 | 0,637456 | 1,030452 | 1,290365 | 2,142445 | 2,707445 | 2,989108 |

**t Test**

RSF-CON

Assuming unequal variances

| Difference | -0,21676 | t Ratio | -0,87851 |
| --- | --- | --- | --- |
| Std Err Dif | 0,24674 | DF | 38,96386 |
| Upper CL Dif | 0,28233 | Prob > |t| | 0,3851 |
| Lower CL Dif | -0,71584 | Prob > t | 0,8075 |
| Confidence | 0,95 | Prob < t | 0,1925 |

**Oneway Analysis of Valeric acid By Treatment**

**Quantiles**

| **Level** | **Minimum** | **10%** | **25%** | **Median** | **75%** | **90%** | **Maximum** |
| --- | --- | --- | --- | --- | --- | --- | --- |
| CON | 0,607952 | 0,941482 | 1,041971 | 1,243256 | 1,483666 | 2,147598 | 2,637499 |
| RSF | 0,204372 | 0,768883 | 0,973573 | 1,350592 | 1,718601 | 2,450642 | 2,612917 |

**t Test**

RSF-CON

Assuming unequal variances

| Difference | 0,06869 | t Ratio | 0,416313 |
| --- | --- | --- | --- |
| Std Err Dif | 0,16500 | DF | 37,80187 |
| Upper CL Dif | 0,40279 | Prob > |t| | 0,6795 |
| Lower CL Dif | -0,26540 | Prob > t | 0,3398 |
| Confidence | 0,95 | Prob < t | 0,6602 |
